# Lectures on Lithotomy, Delivered at the New York Hospital, December, 1837

**Published:** 1839-04

**Authors:** 


### Art. XI.

*Lectures on Lithotomy, delivered at the New York Hospital, December,* 1837. By Alexander H. Stevens, m.d, Surgeon of the New York Hospital, and Emeritus Professor of Clinical Surgery.—*New York,* 1838. 8vo. pp. 93. 4 Plates.

The small publication which bears the above title is divided into two sections or lectures. The first includes the anatomy of the parts concerned in the operation of lithotomy, together with a comparison between the bilateral section, as recommended by Dupuytren, and the unilateral section, as commonly practised in this country.

As we consider that Dr. Stevens has been far from successful in his endeavours to elucidate the anatomy of the different structures which are situated between the cutaneous surface of the perineum and the neck of the bladder, and still less fortunate in his attempts to illustrate them by plates, which indeed are lithographic monstrosities in their way, we shall not follow him through this part of the subject. Neither do we feel ourselves called upon to lay before our readers his remarks respecting the relative advantages of the unilateral and bilateral sections through the perineum and prostate gland, as they will be found almost verbatim in the posthumous work of Dupuytren, on the same subject. Dr. Stevens follows the same train of argument as this great surgeon, to whose views and opinions he professes himself a decided convert.

In the Second Volume of the British and Foreign Medical Review, (p. 99), we gave a full analysis of Dupuytren's work on the "Nouvelle Méthode," or "bilateral section;" and, as we conceive, succeeded in showing how inefficient it was to overcome or do away with those difficulties which it was intended to obviate. We then presented to our readers, as the most conclusive evidence on the subject, as the best commentary on the work in question, the tables of mortality published in France and in England: these proved, in the first place, that the number of patients who sunk after the operation for stone was much greater in France than in this country; and, again, that the success of the distinguished operator himself, under his new method, was far below the general average success of his countrymen; in fact, that more patients have died after the operation of the bilateral section than at any other period or under any new mode of removing the stone from the bladder.

Our present remarks will be confined to a few observations on a new instrument, or bisector, which Dr. Stevens has invented, and which, indeed, forms the *argument* of the pamphlet now before us.

Dr. Stevens describes his new lithotome as follows:

"I venture to offer to the profession a new instrument for the bilateral section of the prostate. In form it resembles a large olive, with a beak at the extremity, with cutting edges at the sides parallel to its longest axis, and with a straight handle. The grooved staff employed in connexion with this instrument is as wide as the urethra will admit, and the groove gradually terminates as it approaches the end of the staff." (p. 53.)

The urethra is opened by a crescentic incision carried across the perineum, with its concavity facing towards the anus, and terminating on either side between the anus and the tuberosity of the ischium. The lithotome is then introduced into the groove of the staff and pushed on into the bladder, dividing the prostate on each side of the urethra.

It will be seen that the method recommended by Dr. Stevens is essentially the bilateral operation of Dupuytren, with a single modification; viz. that, in the one case, the division of the prostate is effected by the entrance of the lithotome into the bladder; in the other, by the withdrawal of the bistouri cachee from the viscus. We consider the alteration made by Dr. S. as a decided improvement, inasmuch as the greater simplicity in the form of the instrument is calculated to facilitate its use, and to diminish the risk which, more especially in inexperienced hands, must always attend the employment of a complicated apparatus. Beyond this, the operation does not differ in the slightest degree from that practised by M. Dupuytren, and is obnoxious to precisely the same objections which we brought forward and explained at considerable length in the article referred to; and which, we conceive, were fully proved by the extraordinary number of fatal cases which followed the use of the double bistouri cachée. In justice to Dr. Stevens, we shall quote his own words as regards the advantages of his plan, and at the same time offer a few observations in reference to the accuracy of his deductions.

"The advantages in the use of this instrument are, 1. That the circular form of a transverse section, gives an opening through the gland of three diameters instead of two, as when a flat instrument is employed: thus it is not necessary to carry the incision so far laterally to obtain an opening of given dimensions; and hence there is less likelihood of hemorrhage from injuring the plexus of vessels that surrounds the prostate." (p. 53.)

As we are by no means certain that we understand Dr. S.'s meaning as to the gain of these diameters in his section through the prostate, we shall refrain from making any observations on the foregoing paragraph which is certainly somewhat obscure.

"2. The prostate is cut horizontally, and though not absolutely, yet for all practical purposes in its greatest diameter." (p. 54.)

We will not cavil at the ambiguity of the language or the equivocation contained in the sentence just quoted. If we rightly understand the distinction attempted to be drawn between the terms "absolutely" and "for all practical purposes," it is intended to imply that a section through each side of the prostate on a level with the urethra (for such must be the line of the knife) produces an opening into the bladder which is best adapted for the extraction of a calculus under circumstances which render the lateral operation difficult and dangerous. We do not mean to deny that a tolerably efficient opening may be secured by the instrument in question, but as the urethra, instead of passing through the centre of the prostate, approaches much nearer to its anterior or pubic aspect, where it is often but thinly covered by the substance of the gland; it is quite impossible that the incision adopted by Dr. Stevens can divide the organ, either absolutely or practically, in its greatest diameter.

"3. The rectum is pushed back by the convexity of the posterior part of the instrument." (p. 54.)

As the rectum cannot, by any possibility, sustain injury during the introduction of the lithotome into the bladder, the advantage just cited dwindles down into comparative insignificance. Indeed, if the safety of the intestine is liable to be compromised during any period of the operation for stone, it is during the first incisions through the perineum, by which the urethra is exposed: no risk is sustained in the subsequent section through the prostate, in whatever way the section may be performed. Upon the whole, the rectum is more likely to be wounded by a transverse semilunar incision through the perineum than by the oblique incision of the lateral operation.

"4. As the prostate is stretched transversely across the instrument, the section is made by a clean cut, and with so little resistance that the instrument does not, like ordinary gorgets, require to be thrust in with force, but may be passed lightly along until the section is completed. Thus there is less danger of wounding the fundus of the bladder by a sudden cessation of resistance from the parts divided; they are, in fact, divided without force."

"5. The easy division of the prostate obviates the dangers of tearing the cellular tissue which connects the anterior surface of the bladder to the posterior wall of the ossa pubis." (p. 54.)

We willingly concede to Dr. Stevens the value of these advantages, when contrasted with the violence necessary for the introduction of the gorget; but his instrument presents, in this respect, no superiority over the knife. The scalpel employed by Mr. Hey, and many other eminent operators, in conjunction with the straight staff, both of which Dr. Stevens so strangely, we had almost said ignorantly, designates as "awkward instruments," is introduced into the bladder with greater facility and less resistance than any other instrument ever devised for the extraction of calculus.

In the subsequent part of his lecture, Dr. Stevens proceeds to speak of the mode in which the stone is to be extracted from the bladder, and the difficulties which are likely to be incurred in its removal, especially if it exceeds the average size.

The causes of resistance to the passage of a stone are thus enumerated by the author, and his own arguments only serve to strengthen our conviction that the lateral operation as performed by the knife is capable, when circumstances require it, of making a larger and at the same time safer opening for the extraction of a large calculus, than can be accomplished by the double-edged lithotome.

"Among the causes opposing the withdrawal of the stone, the muscular fibres at the neck of the bladder, especially when in a state of spasm, are first to be considered. This cause may be overcome without laceration, by steady gentle traction. The second cause of resistance is the fibrous capsule of the prostate gland. This capsule will yield to a limited extent only; beyond which, if force be applied, it will be torn. The third is the substance of the prostate gland itself: this is more disposed to be lacerated than to be stretched. The fourth obstacle is presented by the transversalis muscle, if its fibres have not been completely divided. In a few words, I consider the resistance to arise from the capsule of the prostate, and the neck of the bladder; from the transversalis muscle when not divided on either side; from the levator ani and its investing fasciæ." (pp. 77-79.)

Now all these causes of difficulty present just so many objections to the bilateral operation for the extraction of large stones, simply because the nature of the instrument and the part of the prostate through which it cuts, are such as to limit the size of the opening to a certain extent, beyond which it cannot be carried. In the bilateral operation the transversus perinei muscle is probably not divided at all. The deep fascia is merely cut across and not separated from its attachment to the ramus of the ischium and pubes: the section through the prostate not only takes place in the narrowest place of the gland, but is necessarily limited to its substance, and cannot be carried beyond the organ without compromising the safety of the surrounding venous plexus, or, what is infinitely more dangerous, laying open the subperitoneal cellular texture, and exposing it to urinary infiltration. The unilateral section, on the other hand, tends to remove the track of the knife from the seat of all this mischief. The preliminary steps to open the urethra ensure the division of the transversus perinei, together with the layers of fascia investing the perineal and levator ani muscles, while the section into the bladder, made either at the introduction or the withdrawal of the knife, is carried through the widest part of the prostate, and, if extended beyond that organ, emerges from its substance, not into the subperitoneal tissue, but below that part of the gland where the adhesion of the pelvic fascia forms a barrier between the peritoneum and the lower outlet of the pelvis. If we consider the cavity of the pelvis to be circumscribed or shut in below by the fascia, which, after lining the surface of the levator ani, becomes adherent to the sides of the prostate and neck of the bladder, in that way suspending the viscus, the cavity is never entered at all by the unilateral operation. The bladder is entered below it, and the barrier of aponeurosis, which separates the knife of the surgeon from the subserous tissues, would not be compromised, even though the incision were extended by the side of the rectum until it reached the sacro-sciatic ligament. The opening into the bladder would communicate with the external wound in the perineum, and with nothing else. It is not the least of the advantages afforded by the lateral operation that the deep perineal fascia is separated from its attachment to the pubis and ischium, not merely cut across; and that the more depending situation of the external wound, as the patient lies on his back, affords a readier exit for the escape of the urine than can be obtained by any other kind of incision.

Dr. Stevens asks, "do the bones of the pelvis ever offer any resistance to the extraction of the stone?" and answers his own question by saying, "in the bilateral operation probably not; but in the lateralized operation, if the incision is not carried as near the rectum, and continued as low down as it may be with safety, some resistance must be caused by them." (p. 79.) We hold precisely a contrary opinion; and conceive that the bones are more likely to afford resistance in the bilateral than in any other method of operation, because the incision is made transversely across the narrowest part of the arch of the pubis, in which it can by any possibility be performed. The matter admits of a simple demonstration. The bladder must be entered and the stone subsequently withdrawn through a triangular space, the sides of which are formed by the rami of the pubis and ischium; the base by a line drawn between the tuberosities of the latter bone, while the apex is represented by the symphysis pubis. The urethra passes through this triangle at a short distance below the apex, consequently in the narrower part of the arch. In the bilateral section the urethra is dilated transversely on each side, the lithotome cutting directly across the arch, the incision extending from the canal towards the ramus of each pubis. It follows, therefore, that the opening for the extraction of the stone cannot exceed the width of that part of the arch which is on a level with the urethra. On the other hand, in the lateral operation, the incision commences from the horizontal level of the bilateral section, and is continued downwards into a space which increases in width, in proportion as the incision may be extended in length. Surely Dr. Stevens must have bewildered himself amongst the twenty different layers and sets of structures which he enumerates as intervening between the perineum and bladder, when he ventured upon an assertion so palpably untenable as that which we have just quoted.

Dr. Stevens does not seem to object greatly to the use of force in the extraction of a calculus. He says,

> "It is certain that very successful operators are in the practice of using at times a great deal of force, enough to draw the patient from the table if not held there; and reasoning, no less than experience, justifies this practice within certain limits. The alternative is, to a great degree, between a wound contused even to disorganization, and an extensive deep slough from urinary infiltration. The former is the minor evil. If the opening of the blades of the forceps indicate a stone of more than two inches in its smallest diameter, he will not be able to remove it by a force equal to the lifting of forty pounds, which I conceive is the utmost which is justifiable." (p. 75.)

We certainly have witnessed a very unjustifiable degree of violence exercised in the extraction of stone, but we by no means agree with the author, that the best and most successful surgeons are in the habit of resorting to such a practice. On the contrary, it is held up as most reprehensible. Perhaps no greater objection can be offered to the use of the bilateral lithotome than the fact, that it places the surgeon on the horns of a dilemma when embarrassed with a calculus of more than ordinary size. It leaves him to the choice of two evils, either to extend his incision beyond the prostate in such a direction as will probably endanger the life of his patient by urinary infiltration, or to use such a degree of force as will contuse the parts even to disorganization, if it does not produce mischief more certainly fatal, by tearing the prostate away from its cellular connexions. Dr. Stevens suggests that, when the stone is too large to be removed with safety, it should be crushed in the bladder by instruments adapted for the purpose, and extracted piecemeal.

Having thus expressed our free opinion respecting the operation recommended by Dr. Stevens, it is but right to add, that he has used his prostatic bisector in two cases; in one of which he succeeded in extracting from a boy eleven years old a very large stone, composed of phosphate of lime, and weighing three ounces. In form, it was a flattened ovoid, measuring six inches and a quarter in its longest and four and a half in its shortest circumference. It was finally extracted with the assistance of a curved lever and the fingers of the operator, after dividing the transversus perinei muscle of the right side, which prevented its exit. Erysipelatous inflammation subsequently took place around the wound, but yielded to remedies, and the boy completely recovered. In the second case the stone was about as big as a pigeon's egg, and was extracted with the finger and curved lever, without any particular difficulty. The child was only six years old, and suffered much after the operation from fever and constitutional irritation, but ultimately got quite well.

We cannot conclude this article without making a remark on the lithographic plate, No. 3, which accompanies the book, and which is intended to illustrate the section of the prostate gland in Dr. Stevens's operation. We should be unwilling to impute a want of candour and a wish to deceive the superficial observer; but the pencil of the artist has evidently been biassed by the opinions of the surgeon. We consider that this plate by no means exhibits a fair and accurate view of the parts connected with the operation of lithotomy. The position of the prostate, the situation of the urethra, and the whole arrangement of the sketch are inaccurate, inasmuch as they are warped in favour of the views of the operator. The dotted lines, which mark the cutting track of the lithotome through the prostate, are irreconcileable with the shape of the instrument, as depicted in a previous plate; as the two horizontal blades there indicated cannot by any possibility produce an incision which shall pass downwards and outwards from each side of the urethra.

We feel convinced that Dr. Stevens has not formed an accurate conception of the manner in which the lateral operation is commonly performed in this country, nor of the facilities which are offered by the use of the knife in making a large incision into the bladder. We gather from the publication before us, that his ideas and opinions on the lateral section refer more to the method in which it has been practised in France by means of the lithotome cachée. The bisector invented by Dr. Stevens is certainly an improvement on the double lithotome of Dupuytren, and it also possesses the recommendation of being a safe instrument; but it ought not to supersede the use of the knife, which is all-powerful and efficacious when placed in the hands of a skilful surgeon.

